# Determination of the effects of cinnamon bark fractions on *Candida albicans* and oral epithelial cells

**DOI:** 10.1186/s12906-019-2730-2

**Published:** 2019-11-08

**Authors:** Marie-Pier Veilleux, Daniel Grenier

**Affiliations:** Oral Ecology Research Group, Faculty of Dentistry, Université Laval, 2420 Rue de la Terrasse, Quebec City, QC, G1V 0A6 Canada

**Keywords:** Biofilm, *Candida albicans*, Cinnamon, Essential oil, Epithelial cells, Polyphenols

## Abstract

**Background:**

*Candida albicans* is an opportunistic pathogen that causes oral candidiasis and denture stomatitis. It has also been reported to infect oral mucositis lesions in patients who suffer from cancer affecting the head and neck and who receive chemotherapy and radiotherapy treatments. This study aimed to investigate the effects of two cinnamon bark fractions, i.e., an essential oil and an aqueous extract enriched in proanthocyanidins (Cinnulin PF®) on growth, biofilm formation, and adherence properties of *C. albicans* as well as on oral epithelial cells (barrier integrity, inflammatory response).

**Methods:**

A microplate dilution assay was used to determine antifungal and anti-biofilm properties. A fluorescent assay was used to determine *C. albicans* adherence to oral epithelial cells. Cytotoxicity toward oral epithelial cells was assessed by determination of cell metabolic activity. Tight junction integrity of gingival keratinocytes was assessed by determination of transepithelial electrical resistance. IL-6 and IL-8 secretion by TNFα-stimulated oral epithelial cells was quantified by ELISA.

**Results:**

While Cinnulin PF® did not reduce *C. albicans* growth, the cinnamon bark oil exhibited high antifungal activity with minimum inhibitory concentrations and minimum fungicidal concentrations in the range of 0.039 to 0.078%. The cinnamon oil was also active against a pre-formed *C. albicans* biofilm. Interestingly, Cinnulin PF® prevented biofilm formation by *C. albicans* and attenuated its adherence to oral epithelial cells. At their effective concentrations, the cinnamon oil and the Cinnulin PF® displayed no significant cytotoxicity against oral epithelial cells. In an in vitro model, both cinnamon fractions reinforced the integrity of the oral epithelial barrier. Lastly, Cinnulin PF® inhibited the secretion of interleukin-6 and interleukin-8 by oral epithelial cells stimulated with TNF-α.

**Conclusion:**

By their ability to attenuate growth, biofilm formation and adherence property of *C. albicans*, to reinforce the epithelial barrier function, and to exert anti-inflammatory properties the two cinnamon fractions (essential oil, Cinnulin PF®) investigated in the present study may be promising agents for treating oral infections involving *C. albicans*.

## Background

*Candida albicans* is a commensal fungus that colonizes oral mucosal surfaces and that is normally harmless in healthy individuals as it is maintained at low levels by specific and non-specific salivary and mucosal defense mechanisms as well as by competitive inhibition by oral bacteria [1]. However, under certain circumstances, this opportunistic microorganism can cause a superficial infection called candidiasis. Oral candidiasis is characterized by the appearance of white plaques on inflamed and red mucosa (inner cheeks, tongue, throat) and by pain when eating or swallowing [1, 2]. If the infection becomes invasive, which can occur in immunologically and medically compromised individuals, it can cause septicemia leading to organ failure and eventually death [3]. *C. albicans* has also been reported to infect oral mucositis lesions [4, 5], causing inflammation of the oropharyngeal mucosa [6, 7]. Patients who suffer from cancer affecting the head and neck and who receive chemotherapy and radiotherapy treatments are almost all affected by oral mucositis [6, 7].

*C. albicans* produces several virulence factors that play critical roles in the pathogenic process leading to superficial or systemic infections [8]. The cell surface adhesins of *C. albicans* allow initial adhesion to oral epithelial cells, a key step prior to subsequent tissue invasion and damage [8–10]. *C. albicans* can form biofilms on biotic and abiotic oral surfaces; this increases the resistance of the fungus to antimicrobial agents and the host immune system [2, 11, 12]. Additional virulence factors produced by *C. albicans* include its ability to switch from the yeast form to an invasive hyphae morphotype and to secrete proteolytic and lipolytic enzymes [8]. These pathogenic determinants may be potential targets for new antifungal agents that may limit the appearance of strains resistant to conventional antifungals.

Despite the availability of antifungal agents to treat *C. albicans*-associated oral infections, treatment failures are increasingly common due to the emergence of resistant strains [13–15]. Given this, investigations of the antifungal potential of new molecules are highly relevant. In recent years, plant-derived compounds with antifungal potential have attracted the interest of researchers [16]. Cinnamon, a spice derived from the inner bark of the cinnamon tree, has been reported to possess a number of therapeutic properties, including antimicrobial activity [17, 18]. In the present study, we investigated the effects of two cinnamon bark fractions, an essential oil and an aqueous extract enriched in proanthocyanidins, on the growth, biofilm formation, and adherence properties of *C. albicans*. In addition, an oral epithelial cell model was used to study the effects of the two fractions on the integrity of the epithelial barrier and the host inflammatory response.

## Methods

### Source of cinnamon fractions

A cinnamon extract commercialized as Cinnulin PF® (Lot #: CNCP 1604003) was kindly provided by IN Ingredients Inc. (Spring Hill, TN, USA). The aqueous extract, which was prepared from the bark of *Cinnamomum burmannii*, contains 531.9 mg/g of proanthocyanidins according to the datasheet provided by the company. Cinnamon bark is relatively unusual as it contains proanthocyanidins with a high number of A-type bonds [19]. A 20 mg/mL stock solution of the extract was prepared in 50% (v/v) dimethylsulfoxide and was sterilized by filtration (0.22-μm pore size). Carrier solvent was used as a control in all assays. A cinnamon bark essential oil (Lot #: BHC09A4) extracted from *Cinnamomum verum,* was purchased from Hunzaroma (Longueuil, QC, Canada). The chromatographic analysis performed by the company showed that the cinnamon oil contained cinnamaldehyde (71.35% [v/v]), eugenol (6.18%), linolol (6.02), β-caryophyllene (6.02%), cinnamyle acetate (4.04%), benzyle benzoate (0.96%), p-cymene (0.56%), and 1,8-cineol (0.55%).

### *C. albicans* and culture conditions

*C. albicans* ATCC 28366 (reference strain) and LAM-1 (clinical strain from a case of systemic candidiasis) were cultivated in Sabouraud dextrose medium (BBL Microbiology Systems, Cockeysville, MD, USA) at pH 7 and 37 °C.

### Determination of the minimum inhibitory and minimum fungicidal concentrations

The minimum inhibitory concentration (MIC) and minimum fungicidal concentration (MFC) were determined using a microplate dilution assay. To determine the MIC value, a 24-h culture of *C. albicans* was diluted in fresh culture medium (Sabouraud dextrose medium) to an optical density at 660 nm (OD_660_) of 0.2 corresponding to a 1 McFarland standard. Aliquots (100 μL) of *C. albicans* were added to an equal volume of serial dilutions in culture medium of cinnamon oil (1.25 to 0.0195%) or Cinnulin PF® (1000 to 62.5 μg/mL) in 96-well microplates. Wells without *C. albicans* or without the cinnamon fractions were used as controls. When testing the cinnamon oil, the microplate was covered with an adhesive film to avoid evaporation of the volatile compounds. After an incubation at 37 °C for 24 h (stationary growth phase), growth was monitored by recording the OD_660_ using a microplate reader (Bio-Rad Laboratories, Mississauga, ON, Canada). The MIC value corresponded to the lowest concentration of the cinnamon fractions that completely inhibited growth. To determine the MFC, 5 μL from wells showing no visible growth was spotted on Sabouraud dextrose agar plates, which were incubated at 37 °C for 3 days. The MFC value corresponded to the lowest concentration of the cinnamon fractions where no colony formation was observed. The antifungal agent nystatin was used as a reference antifungal. The MFC/MIC ratio was calculated, and a compound or fraction was considered fungicidal when the ratio was ≤4 and fungistatic when the ratio was > 4 [20]. All assays were performed in triplicate to ensure reproducibility.

### Membrane permeability

The ability of the cinnamon oil at MFC to permeabilize the membrane of *C. albicans* ATCC 28366 was evaluated using SYTOX Green dye (Life Technologies Inc., Burlington, ON, Canada), which binds to DNA once the membrane has been compromised. The assay was performed as previously described [21]. The fluorescence resulting from the binding of the dye to DNA was recorded using a Synergy 2 microplate reader (BioTek Instruments, Winooski, VT, USA) every 15 min for 2 h with the excitation wavelength set at 485 nm and the emission wavelength set at 528 nm. A reaction mixture without essential oil was used as a negative control.

### Biofilm formation and killing

The effect of the cinnamon fractions on biofilm formation by *C. albicans* ATCC 28366 was determined by growing microorganisms in Sabouraud dextrose medium in a 96-well plate in the presence of two-fold serial dilutions of the compounds. Following a 24-h incubation at 37 °C, the medium and free-floating microorganisms were removed by aspiration using a 26 g needle, and the wells were washed three times with distilled water. Biofilms were stained with 100 μL of 0.01% crystal violet for 15 min. The wells were then washed three times with distilled water and were dried at 37 °C overnight, after which 100 μL of 75% ethanol (v/v) was added to each well to release the dye from the biofilm. Absorbance at 550 nm (A_550_) was then measured using a microplate reader. The effect of the cinnamon fractions on biofilm formation was also examined by scanning electron microscopy using the protocol previously described by Lagha et al. [22]. Samples were examined using a JEOL JSM6360LV scanning electron microscope operating at 30 kV. The ability of the cinnamon oil to kill a pre-formed *C. albicans* biofilm was also investigated. Biofilms were prepared in a 96-well plate by cultivating *C. albicans* in Sabouraud dextrose medium for 24 h prior to treatment (1 h) with the cinnamon oil at the MFC value. Biofilm viability was then measured with an XTT [2,3-bis(2-methoxy-4-nitro-sulfophenyl)-2H-tetrazolium-5-carboxanilide sodium salt] assay, as described previously [23].

### Epithelial cell culture conditions and viability assays

The human oral epithelial cell line B11, which was kindly provided by S. Groeger (Justus Liebig University Giessen, Germany) and has already been characterized [24], was cultured in keratinocyte serum-free medium (K-SFM; Life Technologies Inc.) supplemented with growth factors (50 μg/mL of bovine pituitary extract and 5 ng/mL of human epidermal growth factor) and 100 μg/mL of penicillin G-streptomycin. The human oral epithelial cell line GMSM-K [25] was kindly provided by V. Murrah (University of North Carolina, Chapel Hill, NC, USA) and was cultivated in Dulbecco’s Modified Eagle’s Medium (DMEM) supplemented with 10% heat-inactivated inactivated fetal bovine serum (FBS) and 100 μg/mL of penicillin G-streptomycin. The cell cultures were incubated at 37 °C in a 5% CO_2_ atmosphere. Epithelial cells (1 × 10^5^ cells in 200 μL) were seeded into the wells of a 96-well tissue culture plate and were cultivated until they reached confluence. The cells were then treated with either Cinnulin PF® (0, 125, 250, 500, 1000 μg/mL) or cinnamon oil (0, 0.0078, 0.0156, 0.0313, 0.0625, 0.125%) in the appropriate culture medium for 24 h. Their viability was then determined using an MTT (3-[4,5-diethylthiazol-2-yl]-2,5diphenyltetrazolium bromide) colorimetric assay according to the manufacturer’s protocol (Roche Diagnostics, Laval, QC, Canada).

### Adherence to epithelial cells

The effect of the cinnamon fractions on the adherence of *C. albicans* ATCC 28366 to oral epithelial cells was assessed using the human GMSM-K cell line. Epithelial cells were seeded (5 × 10^4^ cells/well) in a 96-well clear bottom black microplate (Greiner Bio One, Frickenhausen, Germany) and were incubated at 37 °C in a 5% CO_2_ atmosphere until they reached confluence. The wells were then washed with DMEM-1% heat-inactivated FBS and were blocked with 1% bovine serum albumin (BSA) to prevent non-specific fungal adherence, and the cinnamon fractions diluted in DMEM-1% heat-inactivated FBS medium were added. Wells without the cinnamon fractions were used as controls. In parallel, cells from an overnight culture of *C. albicans* were labeled with fluorescein isothiocyanate (FITC; Sigma-Aldrich Canada Co.) according to a protocol routinely used in our laboratory [26]. FITC-labeled *C. albicans* was added at a multiplicity of infection (MOI) of 100 to wells containing an epithelial cell monolayer (in the absence or presence of the cinnamon fractions). Following an incubation for 4 h at 37 °C, unbound *C. albicans* were aspirated, and the wells were washed three times with 50 mM phosphate-buffered saline (pH 7; PBS). Adhered *C. albicans* were determined by monitoring fluorescence using a Synergy 2 microplate reader with the excitation and emission wavelengths set at 488 and 522 nm, respectively. Adhered FITC-labeled *C. albicans* were also observed using an Olympus FSX100 fluorescence microscope (Olympus Canada Inc., Richmond Hill, ON, Canada).

### Oral epithelial barrier integrity

The effect of the cinnamon fractions on the integrity of the epithelial barrier was assessed using the human B11 cell line described above and the protocol previously described by Ben Lagha and Grenier [27]. Briefly, epithelial cells (3.5 × 10^5^ cells/insert) were seeded in Costar Transwell™ plates with clear polyester membrane inserts (6.5-mm diameter, 0.4-μm pore size; Corning Co., Cambridge, MA, USA). The basolateral and apical compartments were filled with 0.6 mL and 0.1 mL of culture medium, respectively. Following a 3-day incubation to allow the cells to form tight junctions, the conditioned medium was replaced with antibiotic-free K-SFM, and the cells were incubated for a further 16 h. The cinnamon fractions were then added, and the integrity of the epithelial tight junctions was determined by monitoring the transepithelial electrical resistance (TER) using an ohmmeter (EVOM2, World Precision Instruments, Sarasota, FL, USA) after 2 and 4 h of incubation at 37 °C in a 5% CO_2_ atmosphere. Resistance values were calculated in Ohms (Ω)/cm^2^ by multiplying the resistance values by the surface area of the membrane filter. Results are expressed as a percentage of the basal control value measured at time 0 (100% value).

### Secretion of cytokines by oral epithelial cells

The effect of the cinnamon fractions on the secretion of the pro-inflammatory cytokines interleukin 6 (IL-6) and interleukin 8 (IL-8) was investigated using the GMSM-K epithelial cell line. Cells were seeded in a 6-well plate (10^6^ cells/well in 2 mL) and were cultured overnight at 37 °C in a 5% CO_2_ atmosphere to allow cell adhesion. The epithelial cells were pre-treated with the cinnamon fractions for 30 min prior to stimulating them with 1 ng/mL of recombinant human TNF-α (AnaSpec, Fremont, CA, USA). After a 24-h incubation, cell-free supernatants were collected and were stored at − 20 °C until used. Commercial enzyme-linked immunosorbent assay (ELISA) kits (R&D Systems, Minneapolis, MN, USA) were used to quantify IL-6 and IL-8 concentrations according to the manufacturer’s protocols.

### Statistical analysis

Unless indicated otherwise, all assays were performed in triplicate in two independent experiments, and the means ± standard deviations were calculated. Statistical analyses were performed using a one-way analysis of variance with a post hoc Bonferroni multiple comparison (GraphPad Software Inc.; La Jolla, CA, USA). All results were considered statistically significant at *p* < 0.01.

## Results

The antifungal activity of the cinnamon fractions is reported in Table 1. While Cinnulin PF® at concentrations up to 1000 μg/mL did not reduce the growth of either strain of *C. albicans*, the cinnamon bark oil displayed high antifungal activity, with MIC and MFC values in the range of 0.039 to 0.078% (v/v). Nystatin, which was used as a reference antifungal agent, had a MIC of 50 μg/mL and an MFC of 200 μg/mL. Cinnamon oil is fungicidal rather than fungistatic, with an MFC/MIC ratio in the range of 1 to 2.

**Table 1:** Minimum inhibitory concentrations (MIC) and minimum fungicidal concentrations (MFC) of cinnamon fractions against *C. albicans*

	*Candida albicans*					
	ATCC 28366			LAM-1		
Compounds	MIC	MFC	MFC/MIC* Ratio	MIC	MFC	MFC/MIC Ratio
Cinnulin PF® (μg/mL)	> 1000	> 1000	–	> 1000	> 1000	–
Cinnamon bark oil (% [v/v])	0.039	0.078	2	0.078	0.078	1
Nystatin (μg/mL)	50	200	4	50	200	4

* MFC/MIC Ratio: > 4 means fungistatic activity; ≤ 4 means fungicidal activity

**Fig. 1:**
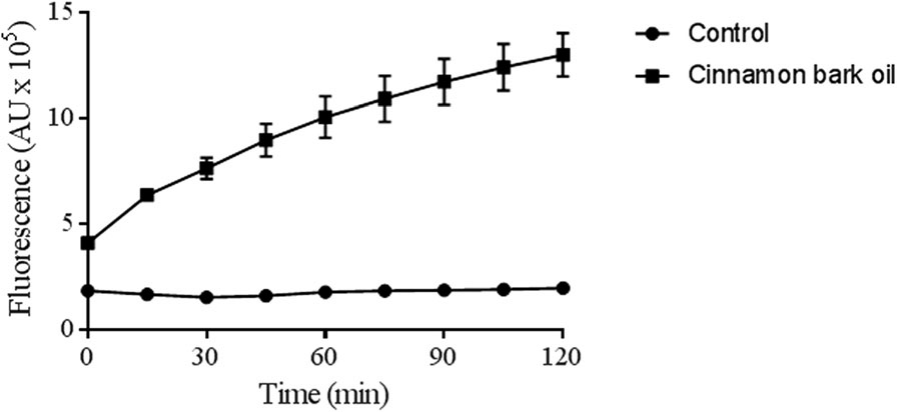
Effect of cinnamon bark oil on the membrane integrity of *C. albicans* ATCC 28266 as determined using SYTOX® Green dye, which penetrates damaged cytoplasmic membranes. *C. albicans* cells were incubated with cinnamon oil at its MFC and fluorescence was recorded for 2 h

SYTOX® Green dye is a fluorescent molecule that penetrates impaired cytoplasmic membranes, binds to DNA, and emits fluorescence. When the *C. albicans* cells were treated with cinnamon oil, a time-dependent increase in fluorescence occurred, suggesting that their membranes had been permeabilized due to the fungicidal activity of the cinnamon oil (Fig. 1). No significant increase in fluorescence occurred in the negative control over the 2-h incubation period. Cinnulin PF®, which had no antimicrobial effect on *C. albicans*, also did not cause an increase in fluorescence (data not shown).

**Fig. 2:**
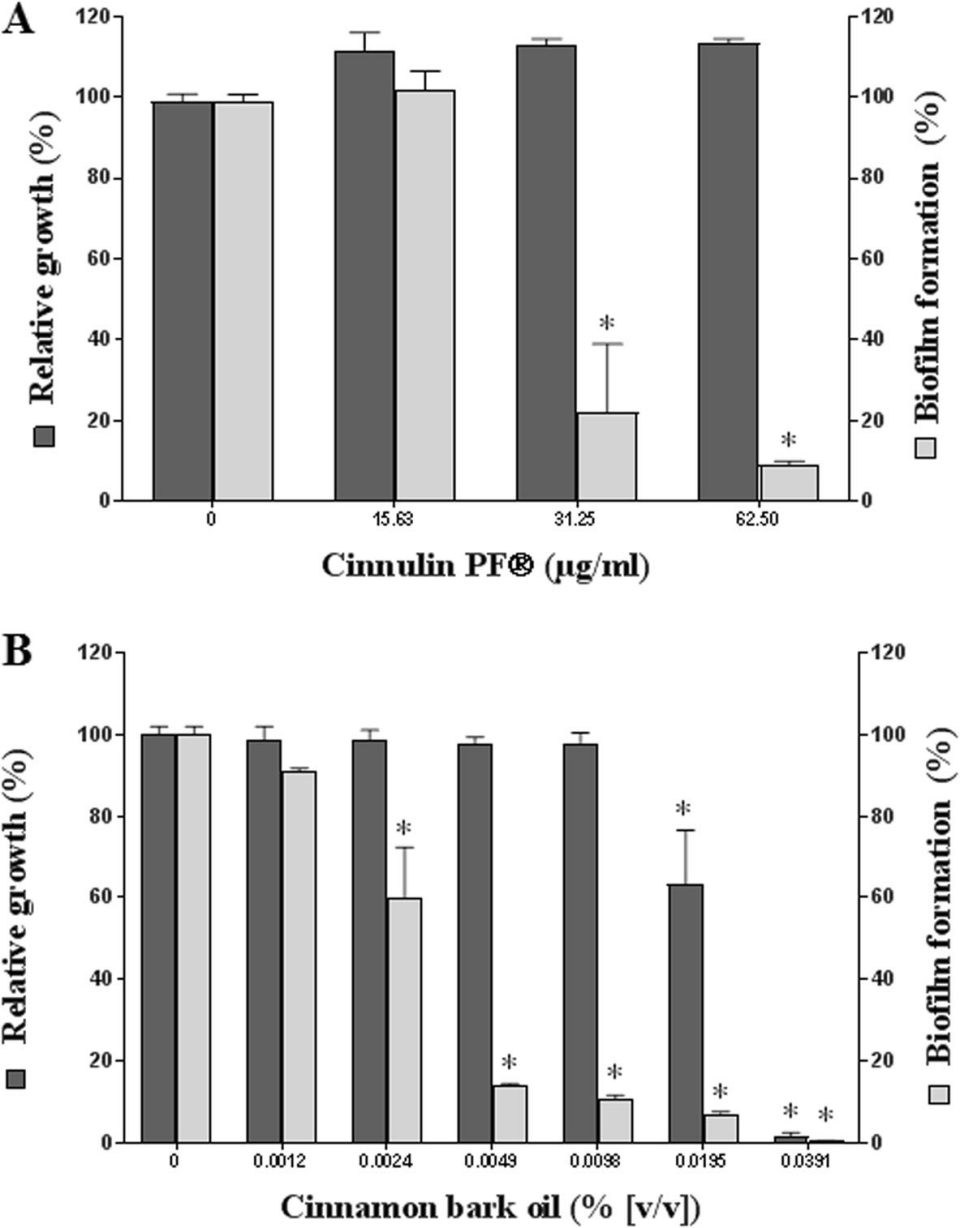
Effect of Cinnulin PF® (panel **a**) and cinnamon bark oil (panel **b**) on the growth and biofilm formation of *C. albicans* ATCC 28266. A value of 100% was assigned to the growth and biofilm obtained in the absence of the cinnamon fractions. Results are expressed as the means ± SD of triplicate assays from two independent experiments. *: significantly different from the control (*p* < 0.01)

**Fig. 3:**
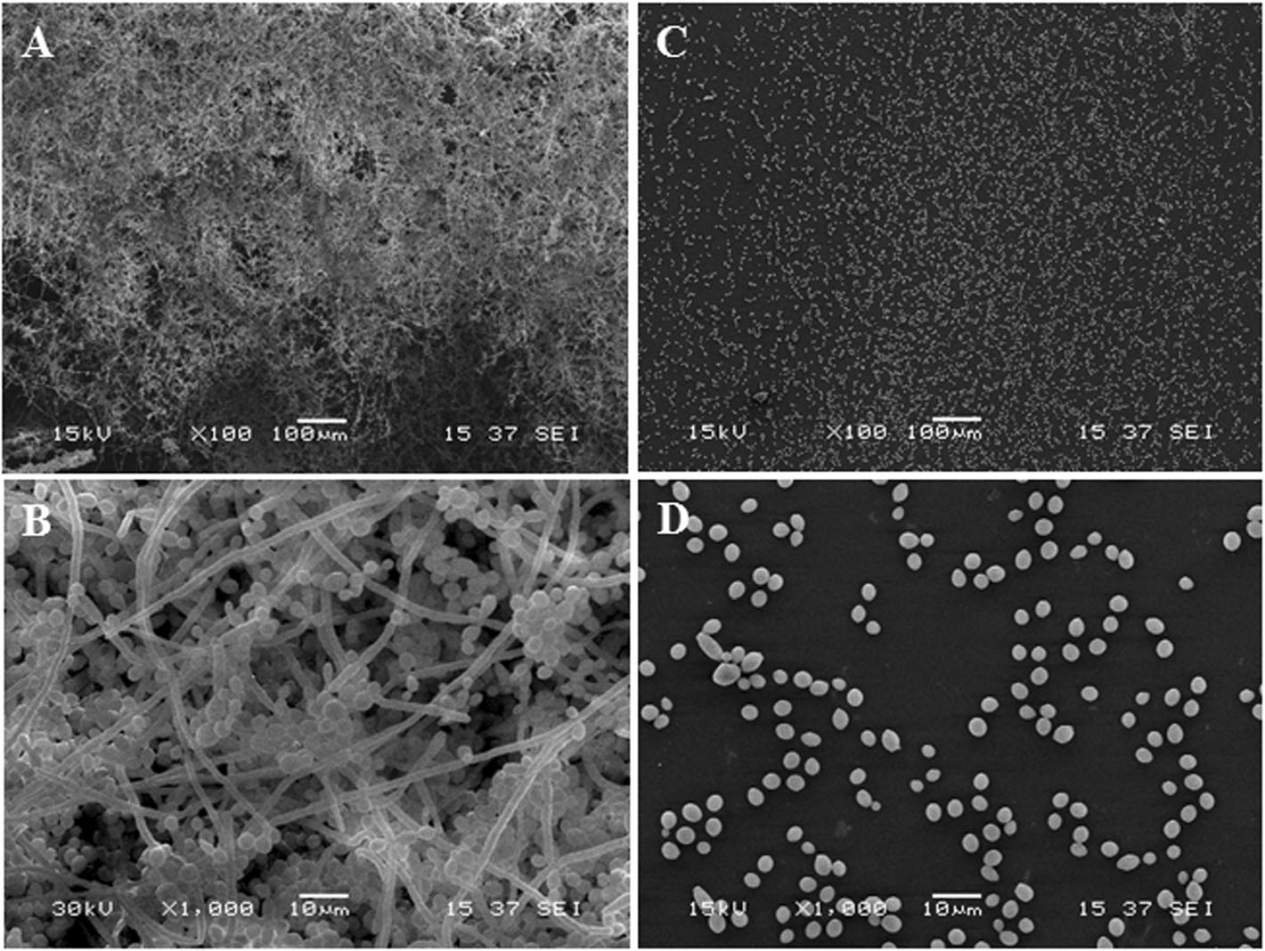
Scanning electron micrographs of biofilms formed by *C. albicans* ATCC 28366 grown in the absence (panels **a** and **b**) or presence of 62.5 μg/mL of Cinnulin PF® (panels C and D). Magnification: 100 X (panels **a** and **c**) and 1000 X (panels **b** and **d**)

The effect of Cinnulin PF® and cinnamon bark oil on biofilm formation by *C. albicans* was then investigated. Although Cinnulin PF® did not reduce the growth of *C. albicans*, it significantly attenuated biofilm formation as determined by crystal violet staining (Fig. 2a). More specifically, at a Cinnulin PF® concentration of 62.5 μg/mL, biofilm formation was reduced by 91%. The effect of Cinnulin PF® on biofilm formation by *C. albicans* was also visualized by scanning electron microscopy. The control biofilm of *C. albicans* appeared dense, and hyphae were an important structural component (Fig. 3a and b). Electron micrographs clearly showed the marked reduction in mature biofilm when *C. albicans* was grown in the presence of 62.5 μg/mL of Cinnulin PF® (Fig. 3c and d). In addition, no hyphae were observed. The cinnamon bark oil also attenuated biofilm formation by *C. albicans* at concentrations that did not inhibit growth. The formation of biofilm was reduced by 86% when *C. albicans* was grown in the presence of 0.0049% cinnamon oil (Fig. 2b).

**Fig. 4:**
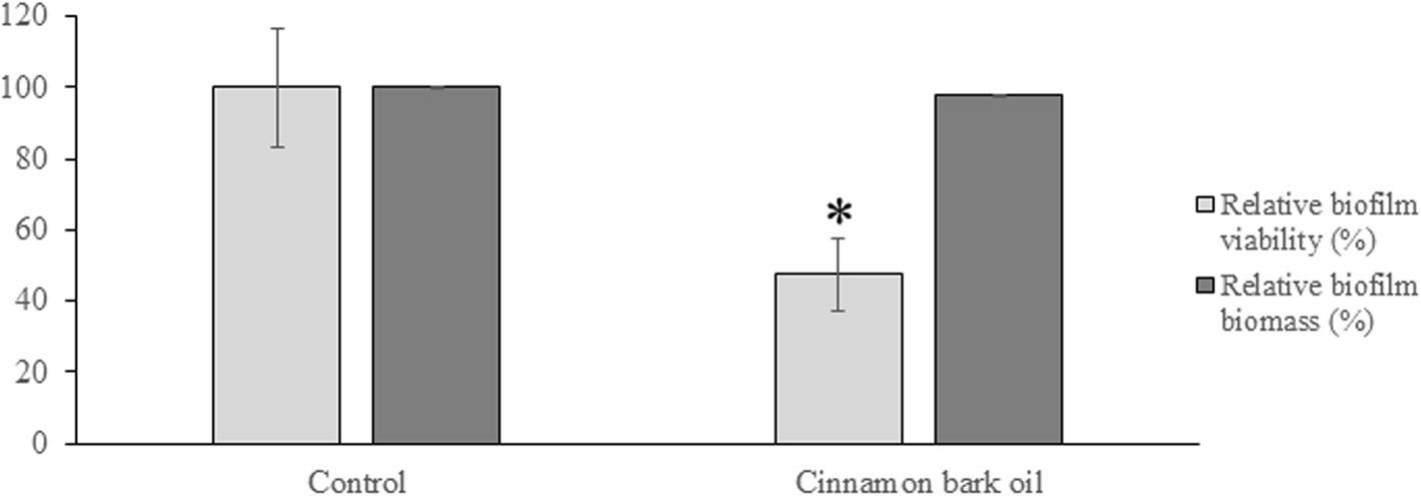
Effect of cinnamon bark oil on the viability and desorption of a *C. albicans* ATCC 28366 biofilm. A pre-formed (24 h) *C. albicans* biofilm was treated for 60 min with cinnamon oil at its MFC, and the residual biomass and viability were measured by crystal violet staining and an XTT assay, respectively. A value of 100% was assigned to the pre-formed biofilm exposed to the cinnamon oil carrier solvent. The assays were performed in triplicate, and the mean ± SD of two independent experiments was calculated. *: significantly different from the control (*p* < 0.01)

Given the fungicidal activity of the cinnamon bark oil, we determined whether it could kill *C. albicans* biofilms. Since Cinnulin PF® did not show any antimicrobial effect against *C. albicans*, it was not tested in this analysis. A 24-h pre-formed *C. albicans* biofilm was treated for 60 min with cinnamon oil at its MFC. Residual viability was determined using an XTT assay that measures metabolic activity. This treatment reduced biofilm viability by 48%, but did not cause any desorption of the biofilm biomass (Fig. 4).

**Fig. 5:**
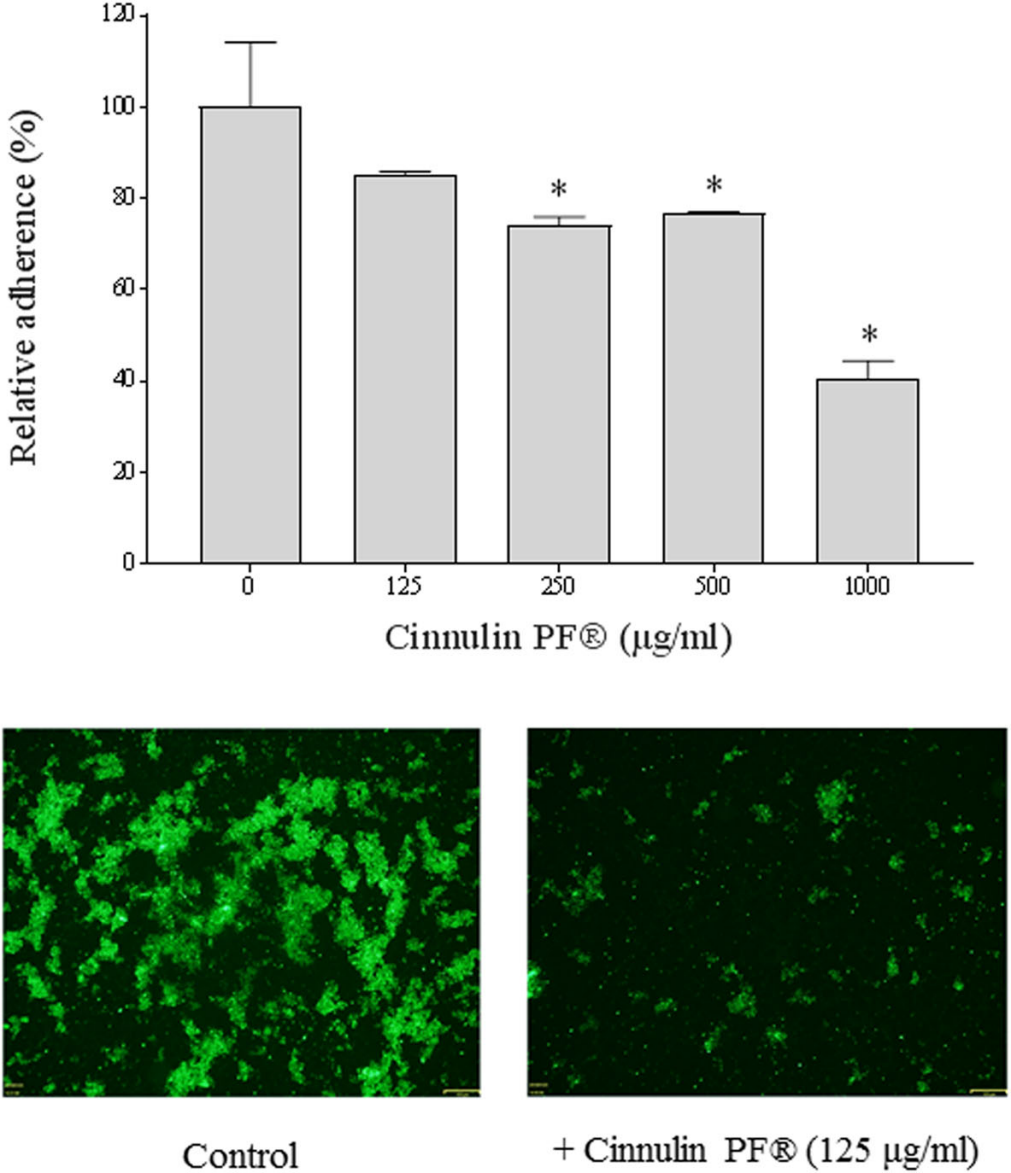
Effect of Cinnulin PF® on the adherence of *C. albicans* ATCC 28366 to GMSM-K oral epithelial cells. Panel A: FITC-labeled *C. albicans* cells adhered to epithelial cells were quantified by measuring fluorescence using a microplate reader. A value of 100% was assigned to *C. albicans* adhered to epithelial cells in the absence of Cinnulin PF®. Results are expressed as the means ± SD of triplicate assays from two independent experiments. *: significantly different from the control (*p* < 0.01). Panel B: Fluorescence micrograph of FITC-labeled *C. albicans* cells adhered to epithelial cells

The effect of the cinnamon fractions on the adherence of *C. albicans* to oral epithelial cells (GMSM-K cell line) was then tested. Cinnulin PF® dose-dependently reduced the adherence of FITC-labeled *C. albicans* to epithelial cells (Fig. 5a). More specifically, in the presence of 1000 μg/mL of Cinnulin PF®, adherence was inhibited by 59%. The ability of Cinnulin PF® to reduce the adherence of *C. albicans* to oral epithelial cells was confirmed by fluorescence microscopy (Fig. 5b). Cinnamon bark oil had no inhibitory effect on the adherence of *C. albicans* to oral epithelial cells (data not shown).

**Fig. 6:**
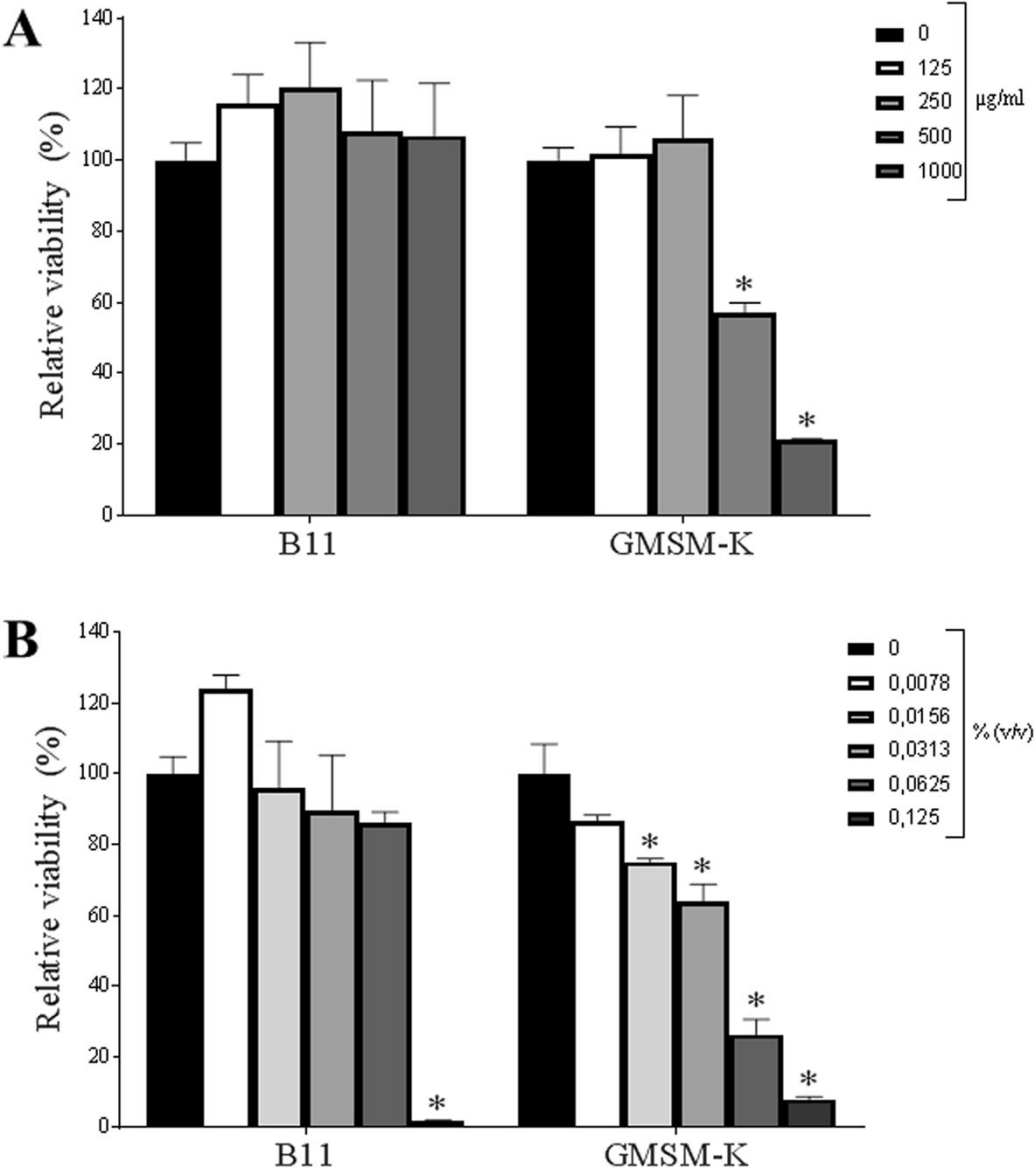
Effect of Cinnulin PF® (panel **a**) and cinnamon bark oil (panel **b**) on the viability of two oral epithelial cell lines (B11 and GMSM-K). The epithelial cells were treated for 16 h with the cinnamon fractions prior to determining cell viability using a colorimetric MTT assay. Results are expressed as the means ± SD of triplicate assays in two independent experiments. *: significantly different from the control (*p* < 0.01)

In order to investigate the biocompatibility of the cinnamon fractions, we tested their effects on the viability of two oral epithelial cell lines. Up to 1000 μg/mL of Cinnulin PF® had no cytotoxic effect on B11 epithelial cells (Fig. 6a). However, 500 μg/mL of Cinnulin PF® reduced the viability of GMSM-K epithelial cells by 42.8%. Treating the B11 and GMSM-K epithelial cell lines with 0.0625% cinnamon bark oil reduced cell viability by 14% (not significant at *p* < 0.01) and 73.8%, respectively (Fig. 6b).

**Fig. 7:**
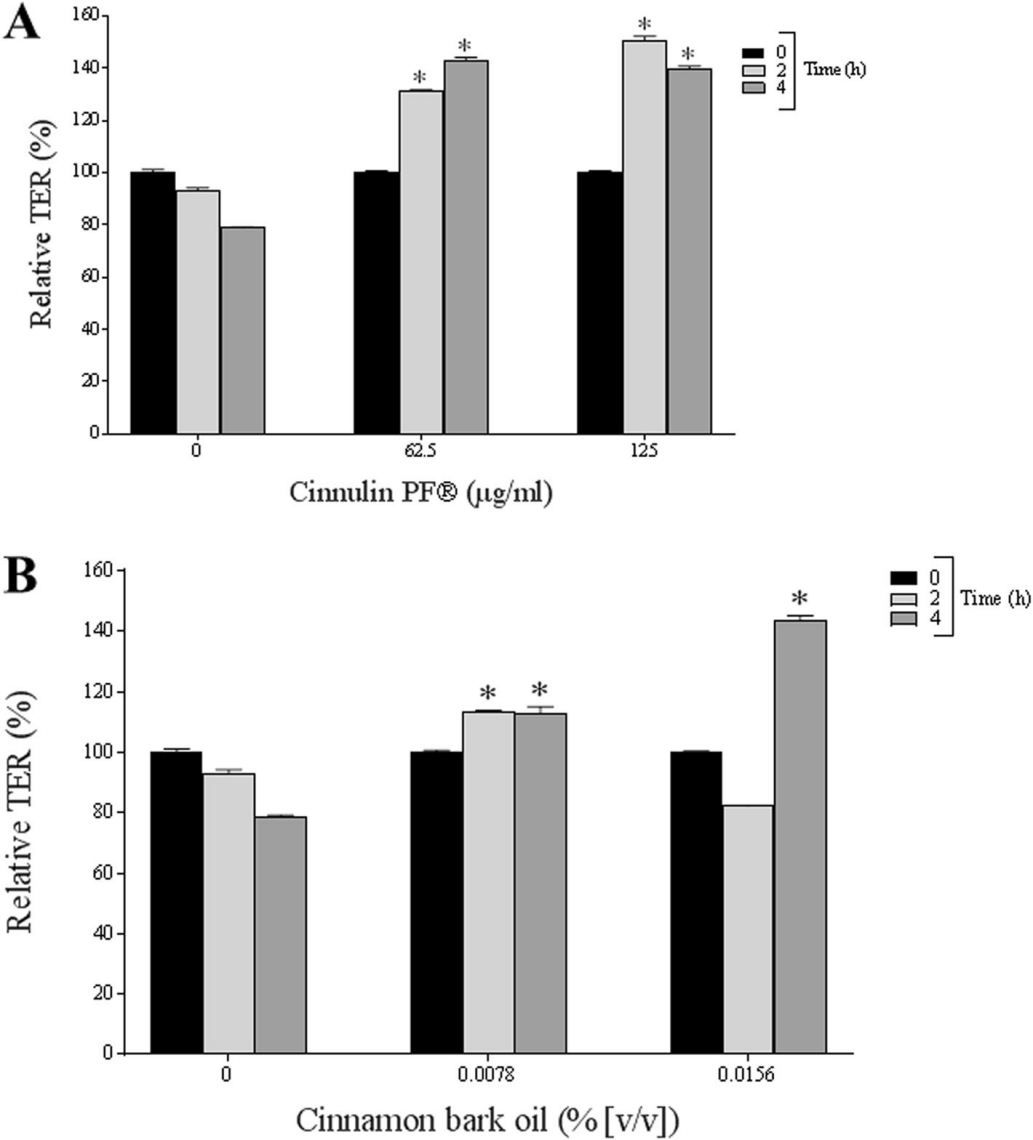
Effect of Cinnulin PF® (panel **a**) and cinnamon bark oil (panel **b**) on the integrity of the epithelial barrier (B11 cell line). The TER values were determined after a 6-h incubation. A value of 100% was assigned to the TER values at time 0. Results are expressed as the means ± SD of triplicate assays. *: significantly different from the control (*p* < 0.01)

We then investigated the effect of Cinnulin PF® and cinnamon bark oil on the integrity of the epithelial barrier by monitoring the TER values of the B11 cell line. After a 4-h incubation, 62.5 μg/mL and 125 μg/mL of Cinnulin PF® time-dependently increased the TER values of the B11 cell line by 42.9 and 39.5%, respectively (Fig. 7), while 0.0156% cinnamon oil increased the TER value by 43.9%.

**Fig. 8:**
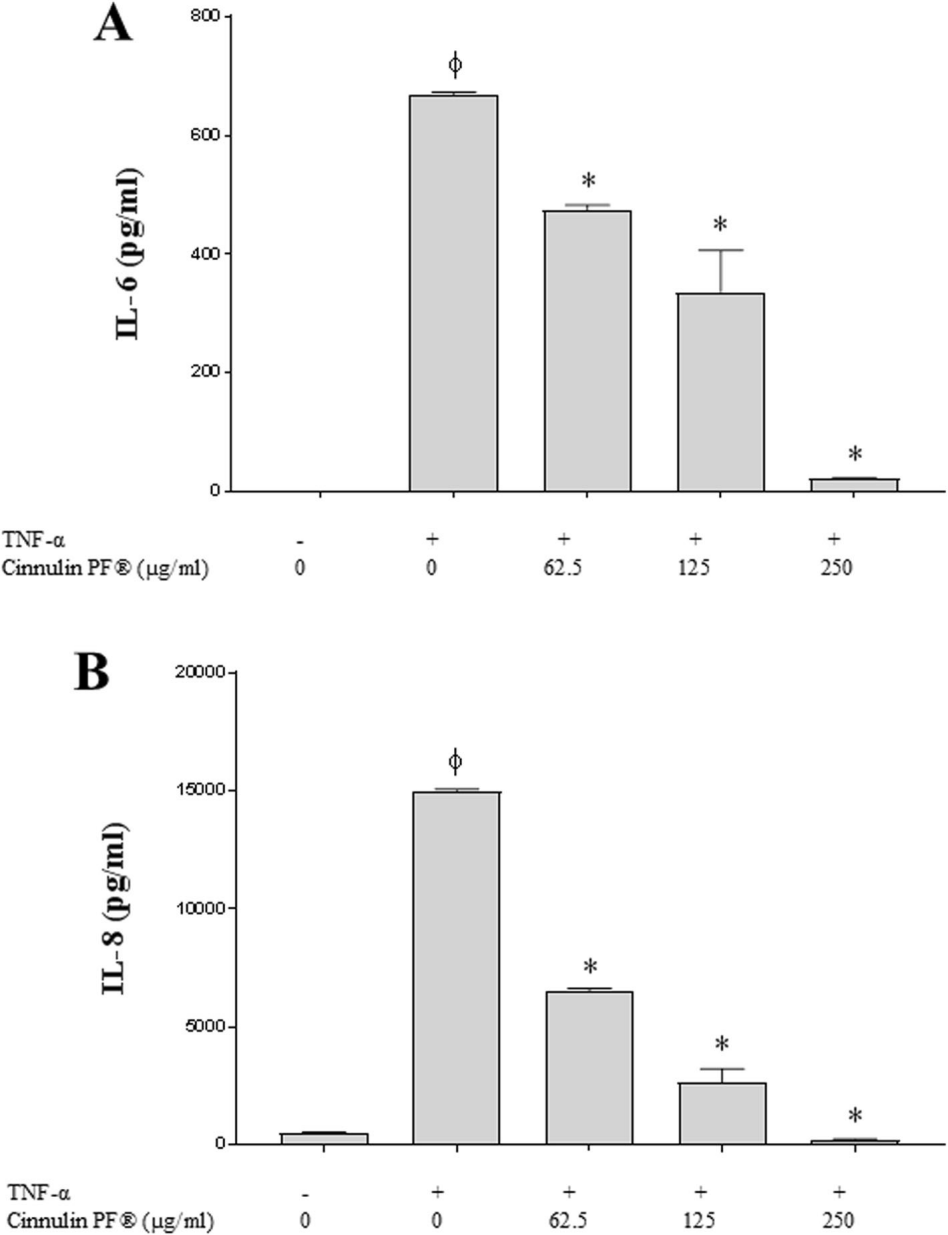
Effect of Cinnulin PF® on TNF-α-induced IL-6 (panel **a**) and IL-8 (panel **b**) secretion by oral epithelial cells (GMSM-K cell line). Results are expressed as the means ± SD of triplicate assays in two independent experiments. *: significantly different from the control (*p* < 0.01)

We assessed the anti-inflammatory properties of Cinnulin PF® using an oral epithelial cell model (GMSM-K cell line) stimulated with TNF-α. Stimulating the epithelial cells with 1 ng/mL of TNF-α induced the secretion of IL-6 (670 pg/mL) and IL-8 (15,008 pg/mL). A 30-min pre-treatment with 62.5 μg/mL of Cinnulin PF® prior to stimulating the epithelial cells with TNF-α reduced the secretion of IL-6 and IL-8 by 29 and 57%, respectively (Fig. 8) while 250 μg/mL of Cinnulin PF® almost totally inhibited the secretion of the two cytokines. The cinnamon bark oil did not reduce the secretion of IL-6 or IL-8 at non-cytotoxic concentrations (≤ 0.0078%; data not shown).

## Discussion

*C. albicans* can be isolated from various sites in humans. It is an opportunistic pathogen and has been associated with superficial and systemic infections, especially in immunologically or medically compromised individuals [3, 11]. *C. albicans* causes oral candidiasis and denture stomatitis, and may also be involved in dental caries, periodontal diseases, and refractory endodontic infections [2]. Ulcerative oral lesions (oral mucositis) resulting from chemotherapy and radiotherapy treatments are susceptible to secondary infections by oral microorganisms, including *C. albicans* [4, 5]. For instance, Belazi et al. [28] isolated *Candida* spp. from oral mucositis lesions in 77% of patients undergoing radiotherapy for head and neck cancer.

*C. albicans* infections can generally be successfully treated with conventional antifungal agents. However, the emergence of resistance to these therapeutic agents is of increasing concern [13–15], which is why investigations of the antifungal potential of new molecules are highly relevant. Plants and their derivatives are an important source of bioactive molecules. Essential oils extracted from different parts of certain plants (leaves, flowers, seeds, bark, etc.) possess numerous therapeutic properties, including antimicrobial activities [29, 30]. Moreover, proanthocyanidins, a family of polyphenols consisting of flavan-3-ol oligomers and polymers, have been proposed as promising molecules for treating oral infections given their anti-adherence and anti-inflammatory properties [31]. The present study was designed to evaluate the effects of two cinnamon fractions, an essential oil and an aqueous extract enriched in proanthocyanidins, on both *C. albicans* (growth, biofilm formation, adherence properties) and oral epithelial cells (barrier integrity, inflammatory response).

We first showed that the growth of *C. albicans* was inhibited by cinnamon bark oil. Cinnulin PF®, had no effect on the growth of *C. albicans*, even at the highest concentration tested (1000 μg/mL). The ability of cinnamon oil to inhibit the growth of several oral microbial pathogens, including *Porphyromonas gingivalis* [32], *Solobacterium moorei* [20], *Streptococcus mutans* [33], and *C. albicans* [34] has been previously reported. Moreover, in a recent study, Essid et al. [34] showed that combining cinnamon oil with the antifungal drug fluconazole provided a synergistic effect against fluconazole-resistant *Candida* strains.

We then explored the mechanism by which the cinnamon bark oil exerts its antifungal effect against *C. albicans*. The ability of cinnamon oil to disrupt the cell membrane was assessed by SYTOX® Green staining, which showed that the antifungal activity of cinnamon oil may be due to its ability to damage the cell membrane. This is in agreement with Essid et al. [34], who reported that cinnamon essential oil inhibits ergosterol biosynthesis in *Candida* species, an effect that may have an impact on the integrity of the fungal membrane by permeabilizing the cell. However, despite the ability of cinnamon oil to cause damage to the cell membrane of *C. albicans*, additional mechanisms that may contribute to its fungicidal effect cannot be ruled out.

*C. albicans* forms biofilms on many oral surfaces, including tooth enamel, oral mucosa, implants, and dentures [1, 2]. *C. albicans* cells embedded in a biofilm are more resistant to mechanical elimination by saliva and to antifungal agents compared with their planktonic counterparts [35–38]. Antimicrobial agents have difficulty penetrating a biofilm, which can reduce their effectiveness. Therapeutic strategies aimed at inhibiting biofilm formation are thus highly relevant. The present study showed that cinnamon bark oil at sub-inhibitory concentrations can inhibit *C. albicans* biofilm formation. Moreover, the treatment of a preformed *C. albicans* biofilm with cinnamon bark oil significantly reduced the viability of the biofilm. A very low concentration of Cinnulin PF® (≥ 31.25 μg/mL) also significantly inhibited the formation of a biofilm by *C. albicans*. These results suggest that Cinnulin PF® may be a promising anti-*C. albicans* agent because it specifically acts on biofilm formation, a critical step of the infectious process. Preliminary assays showed that Cinnulin PF® had no effect on hyphae formation (data not shown). In vivo, biofilm formation by *C. albicans* requires initial adherence to the oral mucosa. Interestingly, Cinnulin PF® significantly attenuated the adherence of *C. albicans* while no such effect was observed with cinnamon oil.

The oral epithelium protects the underlying tissues from microbial invasion and thus actively contributes to the maintenance of oral health [39]. This barrier effect is mediated by the tight junctions that seal the epithelial cells together. We thus investigated the ability of the cinnamon fractions to strengthen the epithelial barrier. Our results demonstrated that electrical resistance increased when the epithelial cells were cultivated in the presence of either cinnamon bark oil or Cinnulin PF®. These results suggest that these cinnamon fractions, by reinforcing the epithelium, may potentially prevent the invasion of the oral mucosa by oral pathogens.

Although the host inflammatory response is key to maintaining oral health, an acute and exacerbated inflammatory reaction as observed in oral candidiasis and oral mucositis may be deleterious by causing tissue damage. More specifically, the development of oral mucositis in patients receiving chemotherapy and radiotherapy treatments involves the stimulation of infiltrating macrophages, resulting in the activation of NF-κB [6, 7]. This process is associated with the secretion of inflammatory cytokines, including TNF-α, that promote inflammation and tissue destruction. In the present study, when epithelial cells were challenged with TNF-α, they secreted a large quantity of IL-6 and IL-8. These two pro-inflammatory cytokines are known to play a critical role for the recruitment and activation of neutrophils and macrophages at the site of infection [40, 41]. However, due to this protecting reaction of the host against fungal pathogens, an accumulation of inflammatory mediators occurs to induce a chronic and persistent inflammation, and ultimately tissue destruction. Therefore, preventing an excessive activation of innate immunoeffectors may be associated with resolution of the inflammatory process. In this study, we showed a dose-dependent inhibitory effect of Cinnulin PF® on TNF-α-induced secretion of IL-6 and IL-8 by oral epithelial cells.

In this study, we showed that the two cinnamon fractions under investigation share a number of common properties (anti-biofilm, tight junction promotion) but also exhibit some distinct features. More specifically, cinnamon essential oil inhibited *C. albicans* growth while Cinnulin PF® attenuated the epithelial cell inflammatory response. Therefore, combining the two cinnamon fractions may be a valuable therapeutic approach for the treatment of *C. albicans* infections through their effects on different targets.

## Conclusion

By their ability to attenuate growth, biofilm formation and adherence property of *C. albicans*, to reinforce the epithelial barrier function, and to attenuate the inflammatory response of epithelial cells, the two cinnamon fractions (essential oil, Cinnulin PF®) investigated in the present study may be promising agents for controlling *C. albicans* infections such as oral candidiasis, denture stomatitis, and *Candida*-infected oral mucositis lesions.

## Data Availability

The datasets used and/or analysed during the current study are available from the corresponding author on reasonable request.

## Abbreviations

DMEM: Dulbecco’s Modified Eagle’s Medium
ELISA: Enzyme-linked immunosorbent assay (ELISA)
FBS: Fetal bovine serum
FITC: Fluorescein isothiocyanate
IL-6: Interleukin-6
IL-8: Interleukin-8
K-SFM: Keratinocyte serum-free medium
MFC: Minimum fungicidal concentration
MIC: Minimum inhibitory concentration
MTT: 3-[4,5-diethylthiazol-2-yl]-2,5diphenyltetrazolium bromide
OD: Optical density
TER: Transepithelial electrical resistance
TNF-α: Tumor necrosis factor-alpha
XTT: 2,3-bis(2-methoxy-4-nitro-sulfophenyl)-2H-tetrazolium-5-carboxanilide sodium salt
